# An investigation of dispositional mindfulness and mood during pregnancy

**DOI:** 10.1186/s12884-019-2416-2

**Published:** 2019-08-01

**Authors:** Adele Krusche, Catherine Crane, Maret Dymond

**Affiliations:** 1 University of Oxford, Oxford, UK; 2 Psychology Department, University of Southampton, Shackleton Building Room 3045, Highfield Campus, University Road, Southampton, Hampshire SO17 1BJ UK

**Keywords:** Dispositional mindfulness, Pregnancy, Prenatal mood, Stress, Labour worry

## Abstract

**Background:**

Mindfulness courses are being offered to numerous groups and while a large body of research has investigated links between dispositional mindfulness and mood, few studies have reported this relationship during pregnancy. The aim of this study was to investigate this relationship in pregnant women to offer insight into whether an intervention which may plausibly increase dispositional mindfulness would be beneficial for this population.

**Methods:**

A cross-sectional analysis was conducted to explore potential relationships between measures of mindfulness and general and pregnancy-specific mood. A sample of pregnant women *(n* = 363) was recruited using online advertising and community-based recruitment and asked to complete a number of questionnaires online.

**Results:**

Overall, higher levels of mindfulness were associated with improved levels of general and pregnancy-related mood in pregnant women. Controlling for general stress and anxiety, higher scores for mindfulness in (psychologically) healthy women were associated with lower levels of pregnancy-related depression, distress and labour worry but this relationship was not apparent in those with current mental health problems. In participants without children, higher mindfulness levels were related to lower levels of pregnancy-related distress.

**Conclusions:**

These results suggest a promising relationship between dispositional mindfulness and mood though it varies depending on background and current problems. More research is needed, but this paper represents a first step in examining the potential of mindfulness courses for pregnant women. Increasing mindfulness, and therefore completing mindfulness-based courses, is potentially beneficial for improvements in mood during pregnancy.

**Electronic supplementary material:**

The online version of this article (10.1186/s12884-019-2416-2) contains supplementary material, which is available to authorized users.

## Background

Existing research evaluating mindfulness and pregnancy has explored the utility of mindfulness courses during pregnancy but the mechanism of change is unclear. Exploration of how mindfulness relates to mood during pregnancy should be conducted to expand the literature and support studies examining the change which takes place during and after mindfulness-based courses but very few studies have examined the relationship between dispositional mindfulness and mood in non-intervention samples. One study found that higher dispositional mindfulness was associated with lower anxiety during pregnancy and less self-regulation problems and negative affect in the 10 month old infant [1]. A further small study [2] found that higher ‘act aware’ dispositional mindfulness (a subscale on the FFMQ [3]) during pregnancy was related to lower postnatal depression and anxiety scores and that as prenatal mindfulness decreased over time, postnatal depression and anxiety scores increased. A recent study found a similar relationship with dispositional mindfulness during pregnancy such that higher levels of mindfulness were related to lower levels of depression and distress [4].

Pregnancy-specific and general anxiety and stress likely reflect different emotional constructs [5–8]. However, research to date has not examined the association between dispositional mindfulness and pregnancy specific measures.

This study aimed to extend previous work by examining the association of dispositional mindfulness with general and pregnancy specific measures in a large sample of pregnant women. The results were examined to evaluate whether increasing dispositional mindfulness during pregnancy may be beneficial, something which mindfulness courses are purported to do [9]. It appears important to elucidate the relationship between dispositional mindfulness and mood during pregnancy to better understand whether, and how, courses offered during this time may be helpful. Individual difference variables such as dispositional mindfulness may have a greater impact on variables such as worry about labour in those who have no prior experiences of childbirth to pattern their beliefs, fears and expectations. Therefore we divided and analysed the sample for those with and without children. In individuals with pre-existing mental health problems, anxiety about pregnancy and labour may reflect underlying psychopathology in addition to situation specific concerns. As such individual differences in mindfulness may play a different, perhaps lesser role in determining distress in these participants. Therefore we divided and analysed the sample in two groups as a function of their pre-existing mental health problems.

## Methods

### Participants

Data was drawn from three samples of expectant mothers: a cross-sectional survey sample investigating various aspects of mindfulness and mood during pregnancy (*n* = 157) and baseline data from two studies exploring the potential of an online mindfulness course for use during pregnancy (*n* = 207). The sample was non-clinical; responses to the questionnaires were expected to vary within the normal range. The minimum age was 18 years. Only participants who completed all questionnaires were included; the data collection website was configured to prevent incomplete pages from being submitted.

### Procedure

Participants were recruited using online advertising, including Facebook, Twitter, motherhood forums and through distribution of information about the project to local community buildings. Participants were directed to a study website where the participant information was displayed and if they wanted to take part, they were directed to sign consent (on the website, by ticking boxes to agree to the consent form statements and entering their name and date) and complete questionnaires using the Online Survey website [10], see Additional file 1 for the Checklist for Reporting Results of Internet E-Surveys [11]. Informed consent was obtained from all individual participants included in the study.

### Measures

A demographic questionnaire included questions about familial and occupational status and the birth (Additional file 2). The following measures were then presented:

#### Dispositional mindfulness

The Five-Facet Mindfulness Questionnaire-15 (FFMQ [3, 12]) measures dispositional mindfulness ranging from 15 to 75. The scale incorporates mindfulness facet subscales: observe; describe; non-judgement; non-reactivity; awareness. FFMQ mindfulness has shown to increase following mindfulness courses [12]. Cronbach’s α for the present study = .75.

#### Perceived stress

The Perceived Stress Scale (PSS [13]) was included in this study to measure general stress and measures how uncontrollable and overwhelming past month events are perceived to have been, ranging from 0 to 40. Cronbach’s α = .78.

#### General anxiety

The General Anxiety Disorder Scale (GAD-7 [14]), measures general anxiety for two preceding weeks and has decreased in a highly anxious pregnant sample following mindfulness courses [15]. Scores range from 0 to 21. Cut-offs are 5, 10 and 15 for mild, moderate and severe anxiety. Cronbach’s α = .90.

#### Perinatal depression

The Edinburgh Postnatal Depression Scale (EPDS [16]) measures levels of depression over the previous week and ranges from 0 to 30. The measure is designed to assess levels of depression during pregnancy and does not include items that might be confounded by physical aspects of pregnancy (e.g. fatigue, sleep disturbance). A score of 9/10 indicates possible depression; 12/13 likely depression. It has been used during the prenatal and postnatal phases [17, 18]. Cronbach’s α = .76.

#### Pregnancy distress

The Tilburg Pregnancy Distress Scale (TPDS [19]) measures pregnancy-related distress for the preceding 7 days and ranges from 0 to 48. A score of more than 17 indicates ‘distressed’. High TPDS distress has been associated with lower levels of dispositional mindfulness in pregnant samples [4]. Cronbach’s α = .82.

#### Worries about labour

Labour worry is said to differ from anxiety and, at a certain level, is a normal part of pregnancy. The Oxford Worries about Labour scale (OWLS [6]) scale has a range of 10–40 with 10 being the highest worry. The scale was created as a retrospective measure of labour worry using common worries from qualitative data and has not previously been used in a sample of pregnant women. The mean labour worry score in a non-clinical sample of new mothers was 25.15 (*SD* 6.72 [6]). Cronbach’s α = .84.

The following measures were completed by the survey study and pilot study samples (*n* = 178).

#### Prenatal distress

The Revised Prenatal Distress Questionnaire (PDQ [7]; PDQ-R [8]) measures pregnancy-related distress ranging from 0 to 34, with questions added at the second and third trimesters. Scores are calculated by dividing the sum by the number of items; normative non-clinical scores are 0.77 overall (SD 0.435 [20]) and 0.72 (SD 0.35), 0.59 (SD 0.32) and 0.55 (0.30) for the three trimesters [8]. Cronbach’s α = .85.

#### Pregnancy-related discomforts

The scale for Pregnancy-Related Discomforts (PRD [21]), asks about the previous week. The ranges are 0–75, 0–65 and 0–65 for the three trimesters respectively. Normative scores for the three trimesters are 36.9, 26.0 and 29.2 [21]. Cronbach’s α = .90 for first trimester discomforts (*n* = 30) and .86 for second trimester discomforts (*n* = 126).

#### Pregnancy experience

The Pregnancy Experience Scale (PES-Brief [22]) has 10 questions for positive and negative experiences (uplifts, hassles) of pregnancy, rated from 0 to 3. Frequency scores are calculated by totalling endorsed questions for uplifts and hassles; previous mean scores are 9.5 for uplifts and 6.5–7.5 for hassles. Intensity scores are calculated by summing the scores for hassles or uplifts and dividing them by the frequency; previous means are 2.4 for uplifts and 1.4 for hassles [22, 23]. Cronbach’s α = .88 for uplifts and .81 for hassles.

### Statistical analysis

A series of Pearson’s correlations were run on mood and mindfulness data to investigate relationships. When exploring pregnancy-specific experience, partial correlations were used to control for general stress (PSS data) and anxiety (GAD-7 data). T-tests were conducted to explore any differences between participants who were currently well and those experiencing mental health difficulties.

## Results

### Data checks

One participant was removed (the first answer was always given), leaving 363 completers. PES hassles showed positive kurtosis (4.609) and the Shapiro Wilk’s test was significant for uplifts and hassles with first and third trimester subsamples (sample split into trimesters for analysis (first *n* = 27, second *n* = 116, third *n* = 13), indicating that trimester analyses conducted using this measure should be non-parametric or bootstrapped.

### Sample characteristics

Approximately half of the sample (56.5%, *n* = 205) had children. Most participants were in their second trimester of pregnancy with 76.6% (*n* = 278) in their second, 12.9% (*n* = 47) in their first and 10.5% (*n* = 38) in their third. Demographic data is shown in Table 1. Most participants were located in the UK, educated to degree level or higher, married or cohabiting and employed.

**Table 1:** Dispositional Mindfulness Study Participant Sociodemographics

Participant Characteristics *n* = 363	%	*n*
Survey Age (participant age taken in brackets: 18–20 - 46-50)	mode: 26–30, 31.1% *n* = 113, mean: 31–35	
UK residents	77.7%	282
Currently married or cohabiting	93.7%	340 (259, 81)
Relationship length (range 1 month - 21 years)	mean: 6.44 years, mode: 3 years	
Educated to degree level	36.6%	133
Educated to postgraduate level	33.9%	123
Currently employed	69.4%	252
Unemployed status-homemaker	21.5%	78
Multiparous	56.5%	205
First trimester	12.9%	47
Second trimester	76.6%	278
Third trimester	10.5%	38
Physical ailments (non-perinatal^a^)	21.8%	79
Mental health problems^b^	14.3%	52
Practice yoga (mode once per week *n* = 38)	19.3%	70
Practice meditation (mode: once per week *n* = 9)	10.2%	37

^b^Including asthma, fibromyalgia, diabetes and various problems with muscles and joints

^b^Most indicating depression, anxiety or comorbid depression and anxiety. Other psychological issues noted included Obsessive Compulsive Disorder, bipolar depressive disorder and Attention Deficit Disorder

### Sample means

Mean scores are presented in Table 2. Sample means were typically above population norms with higher scores for stress, anxiety, TPDS distress (meeting the threshold of 17 for ‘distressed’ [19]) and depression. The smaller sample (made up of survey and pilot samples, *n* = 178) showed moderate pregnancy distress (PDQr [20]), and discomforts [21]. Mean mindfulness was 46.88 (*SD* 9.57), similar to that found with non-clinical pregnant samples previously (48.10, *SD* 7.01) [12].

**Table 2:** Baseline mood scores of total sample *n* = 363

Measure	Mean	*SD*	Research Norms	Publication
PSS Stress	19.50	7.37	11.9–14.7	[24]
GAD-7 Anxiety	7.97	5.31	2.7–3.8	[25]
OWLS Labour Worry	28.17	6.75	25.15	[6]
TPDS Pregnancy Distress	19.27	8.42	10.67	[19]
EPDS Pregnancy Depression	10.72	6.05	7.6	[16, 26]
FFMQ-15 Mindfulness	46.88	9.57	48.10	[12]
Sample *n* = 178				
PDQr Pregnancy Distress	0.73	5.84	0.48–0.71	[20]
PRD 1st Trimester Discomforts *n* = 30	38.97	14.59	36.9	[21]
PRD 2nd Trimester Discomforts *n* = 147	27.81	10.16	26	[21]
PES Frequency of positive experiences	8.28	2.10	6.5–7.5	[22]
PES Intensity score, positive	1.94	0.55	2.4	[22]
PES Frequency of negative experiences	6.93	2.47	9.5	[22]
PES Intensity score, negative	1.52	0.48	1.4	[22]

### Dispositional mindfulness and general mood

Correlations examining the relationship with dispositional mindfulness and general measures of stress, anxiety and depression showed a negative relationship with PSS stress (*r* = −.622, *p* < .001), GAD-7 anxiety (*r* = −.551, *p* < .001) and EPDS Depression, (*r* = −.660, *p* < .001).

### Dispositional mindfulness and pregnancy-related distress

To examine the hypothesis that higher dispositional mindfulness would be associated with lower levels of pregnancy-related distress, correlations were computed between the FFMQ-15, the TPDS (pregnancy distress) and the OWLS (labour worry). There were significant correlations between mindfulness TPDS distress (*r* = −.501, *p* < .001) and OWLS labour worry (*r* = .180, *p <* .005).

### Mindfulness and other aspects of pregnancy experience

Participants from the survey and pilot study samples, *n* = 178, completed several additional measures. These showed that mindfulness was significantly negatively correlated with PDQr distress (*r* = −.430, *p* < .001), first trimester discomfort (*r* = −.447, *p* < .05, *n* = 30), second trimester discomfort (*r* = −.373, *p* < .001, *n* = 147) and the frequency (bootstrapped based on 1000 samples *r* = −.360, *p* < .001, 95% CIs − .469, −.227) and intensity (bootstrapped based on 1000 samples *r* = −.432, *p* < .001, 95% CIs − .550, −.296) of negative pregnancy experiences. There was no correlation between mindfulness and the frequency (bootstrapped *r* = .111, *p =* .139, 95% CIs − .057, .266) or intensity (bootstrapped *r* = .118, *p* .117, 95% CIs − .026, .278) of positive pregnancy experiences.

### Mindfulness and pregnancy experiences

Controlling for general mood; the PSS for perceived stress and GAD-7 for anxiety, partial correlations were re-run with FFMQ mindfulness and pregnancy-related measures (*n* = 363). Mindfulness was still correlated with EPDS depression (*r*_PSS,GAD-7_ = −.325, *p* < .001) and TPDS distress (*r*_PSS,GAD-7_ = −.233, *p* < .001) but not to OWLS labour worry (*r*_PSS,GAD-7_ = .026, *p =* .623).

With the smaller sample (*n* = 178), mindfulness was correlated with PDQr distress (*r*_PSS,GAD-7_ = −.185, *p* < .05) and negative pregnancy experiences, both in frequency (bootstrapped based on 1000 samples *r*_PSS,GAD-7_ = −.235, *p* < .005, 95% CIs − .360, −.094) and intensity (bootstrapped based on 1000 samples *r*_PSS,GAD-7_ = −.176, *p* < .05, 95% CIs − .315, −.016). There was no relationship with first trimester (*r*_PSS,GAD-7_ = −.165, *p =* .400, *n* = 30) or second trimester (*r*_PSS,GAD-7_ = −.067, *p =* .423, *n* = 147) discomforts.

### Dispositional mindfulness and pregnancy-specific mood by parity

See Table 3 for the difference in measures by parity.

**Table 3:** Measure by Parity

Measure	Had prior children				Had no prior children			
	*n*	Mean	*SD*	Range	*n*	Mean	*SD*	Range
PSS stress	205	20.12	7.52	4–36	158	18.70	7.11	1–37
GAD-7 anxiety	205	8.38	5.36	0–21	158	7.44	5.22	0–21
EPDS depression	205	10.94	6.09	0–24	158	10.44	6.00	0–26
TPDS distress	205	18.91	8.32	2–42	158	19.73	8.54	3–40
OWLS labour worry^A^	205	29.85	6.34	10–40	158	25.99	6.65	11–40
PDQr distress^B^	90	10.89	5.68	0–27	88	14.09	5.59	0–27
PRD first trimester discomforts	19	36.58	13.25	17–56	11	43.09	16.50	25–69
PRD second trimester discomforts	71	27.90	10.69	4–57	76	27.72	9.70	9–57
Positive pregnancy experience frequency	90	7.88	2.38	0–10	88	8.68	1.69	3–10
Positive pregnancy experience intensity	90	1.86	0.57	0–3	88	2.02	0.53	1–2.9
Negative pregnancy experience frequency	90	6.57	2.39	0–10	88	7.30	2.49	0–10
Negative pregnancy experience intensity	90	1.56	0.50	0–2.9	88	1.48	0.45	0–2.8
FFMQ Mindfulness	205	46.25	10.13	17–69	158	47.70	8.76	23–70

^A^significant difference between groups t (361) = 5.62, *p* < .001

^B^significant difference between groups t (176) = −3.78, *p* < .001

Partial correlations, controlling for general PSS stress and GAD-7 anxiety were run to examine any difference in those who already had children (*n* = 205) and those who did not (*n* = 158), see Table 4.

**Table 4:** Partial Correlations of Mindfulness and Pregnancy-related Mood, controlling for PSS Stress and GAD-7 Anxiety

Measure	Had prior children			Had no prior children		
	*n*	*r*	*p*	*n*	*r*	*p*
EPDS depression	205	−.443	**.000**	158	−.147	.067
TPDS distress	205	−.204	**.004**	158	−.305	**.000**
OWLS labour worry	205	.011	.880	158	.084	.299
PDQr distress	90	−.074	.495	88	−.357	**.001**
PRD first trimester discomforts	19	−.246	.342	11	.251	.515
PRD second trimester discomforts	71	−.035	.773	76	−.194	.097
Positive pregnancy experience frequency^a^	90	.067	.535	88	−.024	.828
Positive pregnancy experience intensity^a^	90	−.052	.629	88	.129	.235
Negative pregnancy experience frequency^a^	90	−.297	**.005**	88	−.145	.182
Negative pregnancy experience intensity^a^	90	−.207	**.053**	88	−.158	.147

^a^PES analysis bootstrapped based on 1000 samples

These scores represent the values of statistical significance and one near-to significance

In those who had previous children, mindfulness was correlated with EPDS depression (*r*_PSS,GAD-7_ = −.443, *p <* .001) and TPDS distress (*r*_PSS,GAD-7_ = −.204, *p <* .005). OWLS labour worry was not correlated with mindfulness in those with (*r*_PSS,GAD-7_ = .011, *p =* .880) children.

Examining the measures completed by fewer participants (*n* = 178), comparing those with (*n* = 90) and without children (*n* = 88), mindfulness was not correlated with PDQr distress in those with children (*r*_PSS,GAD-7_ = −.074, *p =* .495).

Correlations with mindfulness and pregnancy experience showed that there was still a relationship with the frequency of negative pregnancy experiences and mindfulness in those who already had children (bootstrapped based on 1000 samples *r*_PSS,GAD-7_ = −.297, *p =* .005, 95% CIs − .482, −.116).There was a trend for the intensity of negative pregnancy experiences in those with children (bootstrapped based on 1000 samples *r*_PSS,GAD-7_ = −.207, *p =* .053, 95% CIs − .401, .025). Examining second trimester discomforts, mindfulness was not correlated in those who had children (*r*_PSS,GAD-7_ = −.035, *p =* .773, *n* = 71).

In those without children, EPDS depression was no longer correlated with mindfulness (*r*_PSS,GAD-7_ = −.147, *p =* .067) and was still correlated with TPDS distress (*r*_PSS,GAD-7_ = −.305, *p <* .001). OWLS labour worry was not correlated with mindfulness in those without children (*r*_PSS,GAD-7_ = .084, *p =* .299).

Examining the measures completed by fewer participants (*n* = 178), comparing those with (*n* = 90) and without children (*n* = 88), mindfulness was correlated with PDQr distress (*r*_PSS,GAD-7_ = −.357, *p =* .001) in those who had no previous children.

In those without prior children, there was no longer a relationship between negative pregnancy experience and mindfulness, either frequency (bootstrapped based on 1000 samples *r*_PSS,GAD-7_ = −.145, *p =* .182, 95% CIs − .309, .017) or intensity (bootstrapped based on 1000 samples *r*_PSS,GAD-7_ = −.158, *p =* .147, 95% CIs − .321, .019). Examining second trimester discomforts, mindfulness was not correlated in those who did not have children (*r*_PSS,GAD-7_ = −.194, *p =* .097, *n* = 76).

### Mindfulness, general mood and current mental health problems

Participants were asked whether or not they had current mental health problems and if so, what they were. Of those who did have mental health problems (*n* = 52) a variety of problems were stated including depression (*n* = 22), anxiety (*n* = 15), bipolar depression (*n* = 1) or a mixture of two or more co-morbidities (*n* = 14) including issues such as depression, anxiety, obsessive compulsive disorder, borderline personality disorder, bipolar depression and post-traumatic stress disorder.. A conservative effect size of 0.25 (*f*) [27] was used to determine the t-test power with a sample of 52 compared with 311 healthy participants, using G*Power software [28]. The estimated power for such a test was 80% (*df* 361).

Compared with currently well participants (*n* = 311), participants with mental health issues (*n* = 52) had significantly higher perceived stress, *t* (361) = 5.52, *p* < .001 and anxiety *t* (361) = 6.21, *p* < .001. Dispositional mindfulness was also significantly lower for participants experiencing mental health problems, *t* (361) = − 5.30, *p* < .001. See Table 5.

**Table 5:** Outcomes by Current Mental Health Problems

Measure	Participants with mental health problems (*n* = 52)		Participants without mental health problems (*n* = 311)	
	Mean	*SD*	Mean	*SD*
FFMQ-15 Mindfulness	40.60	9.04	47.93	9.26
PSS Stress	24.52	6.84	18.66	7.13
GAD-7 Anxiety	12.00	5.19	7.30	5.03
EPDS Depression	14.85	5.94	10.04	5.80
TPDS Distress	21.38	9.50	18.91	8.18
OWLS Labour Worry	28.08	7.35	28.19	6.65

Correlations examining the relationship with mindfulness and mood in participants with (*n* = 52) and without (*n* = 311) current mental health problems showed that perceived stress was correlated with mindfulness in the two groups, *r =* −.455, *p* < .005 and *r* = −.612, *p* < .001 respectively and so was anxiety, *r* = −.355, *p* < .05 and *r* = −.536, *p* < .001.

### Mindfulness, pregnancy-related mood and current mental health problems

Participants with mental health issues (*n* = 52) had significantly higher EPDS pregnancy-related depression, *t* (361) = 5.52, *p* < .001 and pregnancy-related TPDS distress *t* (361) = 1.97, *p* = .05 compared with their currently healthy counterparts (*n* = 311).

Correlations split by current mental health problems (*n* = 52) or not (*n* = 311) showed that, in those without problems, mindfulness was correlated with pregnancy-related depression, *r* = −.665, *p* < .001, distress, *r* = −.57, *p* < .001 and labour worry, *r* = .244, *p* < .001. For participants with current problems, mindfulness was correlated with pregnancy-related depression, *r* = −.433, *p* < .005 but not correlated with distress, *r* = −.78, *p* = .58, nor labour worry, *r* = −.140, *p* = .32.

Partial correlations in those with no current mental health problems (*n* = 311), controlling for general mood (PSS stress, & GAD-7 anxiety) showed that mindfulness was correlated with pregnancy-related depression, *r*_(PSS, GAD-7)_ = −.359, *p <* .001 and distress, *r*_(PSS, GAD-7)_ = −.333, *p <* .001 and not with labour worry, *r*_(PSS, GAD-7)_ = .078, *p =* .17. Examining participants with current problems (*n* = 52), mindfulness was not correlated with pregnancy-related depression, *r*_(PSS, GAD-7)_ = −.133, *p* = .36, distress, *r*_(PSS, GAD-7)_ = .136, *p* = .35, nor labour worry, *r*_(PSS, GAD-7)_ = −.192, *p* = .18.

## Discussion and conclusions

The intention of this study was to evaluate the relationship between mood and mindfulness in a cross-sectional analysis of pregnant women to further limited research.

The level of dispositional mindfulness had a significant association with mood such that higher mindfulness scores were related to lower scores of general stress and anxiety and controlling for general mood, pregnancy-related depression, distress and rates of negative pregnancy experiences.

For participants who had children, when accounting for levels of general stress and anxiety, higher mindfulness scores were associated with lower scores of pregnancy-related depression, distress and negative pregnancy experiences. In those without children, higher mindfulness was associated with lower pregnancy-related distress.

Higher levels of mindfulness were related to lower levels of general stress and anxiety whether or not participants had current mental health problems. In those without current problems, when controlling for general stress and anxiety, higher mindfulness scores were associated with lower levels of pregnancy-related depression and distress but for participants who had current mental health problems, there was no relationship.

The current findings show that, in a sample with higher scores of negative mood overall, higher levels of dispositional mindfulness are associated with lower levels of general and pregnancy-related negative mood, but that the background of the participants should be taken into account. The current analysis, being correlational in nature, can only show a relationship and not causality, i.e. it is unclear whether lower levels of mindfulness incur higher levels of stress etc. or that higher levels of stress incur lower levels of mindfulness. While the current findings suggest that offering a mindfulness-based stress reduction course to women during pregnancy may be beneficial, more research should be conducted to investigate the relationship and potential benefits in more detail. This paper presents an initial exploration of how mood and mindfulness relate to each other during pregnancy and is a precursor to future studies investigating mindfulness interventions for pregnant populations.

Research has found that higher dispositional mindfulness during pregnancy was associated with improved mood during and after pregnancy if it was maintained or increased [2]. Potentially, sustaining levels of mindfulness over pregnancy could be beneficial for low mood. Offering a course with mindfulness-based elements, specifically aimed at alleviating low mood during pregnancy, may be most beneficial.

This study has limitations. First, the study is cross-sectional with no follow-up data so it is difficult to posit how these women would have felt later in pregnancy. While splitting the sample by trimester gives an indication of mood during different times, it would be more informative to investigate how mood changes during pregnancy. Second, measures of pregnancy-specific anxiety and stress were not included to limit participant burden; while pregnancy-specific and general anxiety and stress may reflect different emotional constructs, potential differences cannot be currently evaluated because of this omission and it may be helpful to include them in future studies.

This is one of the first studies to explore mood and dispositional mindfulness during pregnancy and as such, is a good precursor to future studies. Proceeding studies should investigate whether mindfulness mediates mood improvement and use this work to improve the rationale and research surrounding the utility of mindfulness courses for use in this population.

## Additional files

Additional file 1: Checklist for Reporting Results of Internet E-Surveys (CHERRIES). (DOCX 15 kb)

Additional file 2: Sociodemographic Questionnaire. (DOCX 20 kb)

## Data Availability

The datasets used during the current study are not publicly available but are available from the corresponding author on reasonable request.
