# Supplementary material for: An investigation of dispositional mindfulness and mood during pregnancy
Source: BMC Pregnancy Childbirth. 2019 Aug 1;19:273. doi: 10.1186/s12884-019-2416-2 (PMC6676599; doi:10.1186/s12884-019-2416-2)
Supplement: Supplementary file 1 — Checklist for Reporting Results of Internet E-Surveys (CHERRIES). (DOCX 15 kb) [file 12884_2019_2416_MOESM1_ESM.docx]

**Additional file 1: Checklist for Reporting Results of Internet E-Surveys (CHERRIES)**

| **Item Category** | Checklist Item | Explanation |
| --- | --- | --- |
| **Design** | | |
| Describe survey design | | Study data was drawn from three participant samples of pregnant women: a cross sectional survey sample (n = 157) and baseline data from two samples enrolled in studies to explore the potential of an online mindfulness course for use during pregnancy (n = 207). The sample was non-clinical. |
| **IRB (Institutional Review Board) approval and informed consent process** | | |
| IRB approval | | All procedures were approved by the institutional research committee, CUREC (MSD-IDREC-C1-2013-016). |
| Informed consent | | Informed consent was obtained from all individual participants included in the study. Participants were informed of the length of time it should take to complete the questionnaires (approximately 25 minutes). This study was conducted as part of a DPhil in Psychiatry. Anonymous data was stored by the primary investigator for the duration of the study and thesis write-up. The purpose of the study was to examine the relationship between various mood outcomes and dispositional mindfulness. |
| Data protection | | Personal and sensitive data were kept separate to the anonymous questionnaire responses and stored on a secure server. |
| **Development and pre-testing** | | |
| Development and testing | | The survey was developed by the primary investigator and consisted of a number of valid and reliable questionnaires. The website/s were tested prior to administering the questionnaires online. |
| **Recruitment process and description of the sample having access to the questionnaire** | | |
| Open survey versus closed survey | | The surveys were open. |
| Contact mode | | The initial contact was made in various ways. Links to the survey were posted on various websites, including Facebook, pregnancy and motherhood forums, community-based posters, and emails. |
| Advertising the survey | | Participants were recruited using online advertising, including Facebook, Twitter, Google Adwords, online motherhood forums and through distribution of information about the project to local workplaces and community buildings. See appendix B for example adverts and information. |
| **Survey administration** | | |
| Web/E-mail | | Web questionnaires. |
| Context | | See appendix B for a list of online advertising methods. Pregnant women were targeted in a variety of ways including online and paper-based advertising (posters). |
| Mandatory/voluntary | | Voluntary survey. |
| Incentives | | For the survey study, participants were entered into a prize draw to win £50 in vouchers. For the online course studies, participants received free access to an online mindfulness-based course. |
| Time/Date | | March 2013-March 2015. |
| Randomization of items or questionnaires | | Items were fixed. |
| Adaptive questioning | | Some questions responded to with certain responses opened up appropriate follow-up questions. For example, answering marital status as 'in a relationship' opened two follow-up questions, one asking if they were living separately, cohabiting, married or other and one asking about the duration of their relationship. |
| Number of Items | | The number of questionnaire items per page varied because a number of different measures were presented. For instance, one measure was made up of seven items and therefore all were presented on one page. In instances where a questionnaire was longer than ten items, the questionnaire was split so that no more than ten items were presented on any one page. |
| Number of screens (pages) | | The questionnaires were presented on approximately fifteen pages. |
| Completeness check | | Pages could only be submitted when all mandatory items were complete. All outcome measure responses were mandatory. Consent form agreement was mandatory. Some sociodemographic items were mandatory and used for screening/cleaning the sample. |
| Review step | | Participants could click Back to change their responses. After the questionnaire was submitted, participants could not access their questionnaires. |
| **Response rates** | | |
| Unique site visitor | | This data was not collected. |
| View rate (Ratio of unique survey visitors/unique site visitors) | | Unavailable. |
| Participation rate (Ratio of unique visitors who agreed to participate/unique first survey page visitors) | | Unavailable. |
| Completion rate (Ratio of users who finished the survey/users who agreed to participate) | | 56.96% (363/639). |
| **Preventing multiple entries from the same individual** | | |
| Cookies used | | Cookies were not saved. Any individual participant responses which matched another were checked for during data cleaning. There were none. As so many items were answered regarding demographics, it is unlikely that one person completed the questionnaires more than once as this would have been captured during cleaning (i.e. participants had to answer questions about their age, relationship status, location, employment status, amongst others). Participants were also asked to enter the last five digits of their mobile number at the end of the questionnaires as an extra step to identify individual responses. |
| IP check | | IP addresses were not saved. |
| Log file analysis | | No other techniques were used. |
| Registration | | No registration was required. |
| **Analysis** | | |
| Handling of incomplete questionnaires | | Only completed pages could be submitted. The sample consists of users who completed the questionnaires in their entirety. Participants who dropped out prior to completing all of the questionnaires were omitted from analysis |
| Questionnaires submitted with an atypical timestamp | | A timestamp was not used. However, when data cleaning, the responses were checked and as such, one participant was removed as they had answered with the first option for each item. |
| Statistical correction | | Weighting procedures were not used. |
