# Supplementary material for: An investigation of dispositional mindfulness and mood during pregnancy
Source: BMC Pregnancy Childbirth. 2019 Aug 1;19:273. doi: 10.1186/s12884-019-2416-2 (PMC6676599; doi:10.1186/s12884-019-2416-2)
Supplement: Supplementary file 2 — Sociodemographic Questionnaire. (DOCX 20 kb) [file 12884_2019_2416_MOESM2_ESM.docx]

**Sociodemographic Questionnaire**

Date of birth____________

Please enter your due date ___________

How many are you expecting? _______

Do you have any children?

- Yes
- No

How many?__

Were there any complications during your previous pregnancies?

- Yes
- No

Please could you decide what these complications were?

Have you had any complications during your current pregnancy?

Please could you describe what these complications are/have been?

Is there anything distressing you about your current pregnancy? (Please describe if yes) (Optional)

Who is providing your care?

- GP
- Community Midwifery
- General Obstetric Clinic
- Maternal Medicine
- Other (please specify):

How many times have you met with your midwife? _________

Where are you planning on giving birth?

- Home
- Freestanding Midwifery Unit
- Alongside Midwifery Unit
- Obstetric Unit
- Hospital
- Other (please specify):

What are your birth preferences? (Select all that apply)

- Elective caesarean
- Drug-free
- Water-birth
- Hypno-birth
- Epidural
- Gas and air
- I'm flexible
- Other (please specify):

Who have you discussed your birth plan with? (Select all that apply)

- No-one
- Partner
- Parent/s
- Other relative/s
- Friends
- Care-provider/Midwife
- Other (please specify):

Who are you planning on having with you at the birth? (Select all that apply)

- No-one
- Partner
- Parent/s
- Other relative/s
- Friends
- Care-provider/Midwife
- Other (please specify):

Marital status:

- Single
- In a relationship
- Separated/Divorced

If in a relationship, are you:

- Living separately
- Cohabiting
- Married

How long (in years) have you been in your current relationship?_______

What is your relationship with the father of your baby?

- Currently together (as above)
- Not together

(If not together) How would you define your previous relationship with he father?

- Never a couple
- Previously together/Separated/Divorced
- Friends
- Other

How long were you in a relationship (in years)? _____

Highest Educational Achievement:

- Postgraduate Degree
- Undergraduate Degree
- A-levels/O-levels (or equivalent)
- GCSEs/GNVQs (or equivalent)
- No exams taken
- Other

What is your current employment status?

- Employed
- Unemployed

What is your job type?

- Administration/Customer Service
- Public Services/Security/Military
- Retail/Sales
- Health Care Worker
- Managerial
- Professional
- Education
- Catering
- Cleaning
- Warehouse/Production
- Tradesman/Construction
- Other. Please specify___________

(If employed)

- Full-time
- Part-time
- Self-employed
- Contract work

(If currently unemployed)

- Looking for a job
- On disability allowance
- Retired
- Student
- Do not work (e.g. stay at home Mum/home-maker)

What is your accommodation?

- Homeowner
- Rented
- Council House
- Living with parents
- Other (please specify)__________

(Optional) What is your household gross income (before tax)?

- £12000 or less
- £12001-£20000
- £20001-£30000
- £30001-£40000
- £40001-£50000
- £50001-£60000
- More than £60000

(Optional) Do you smoke?

- Yes
- No
- Not while I am pregnant

How many cigarettes do you smoke a day?

- Less than 5
- 5-10 per day
- 11-15 per day
- 16-20 per day
- More than 20 per day

(Optional) Are you currently drinking any alcohol?

- Yes
- No

How much alcohol do you drink per week on average?

[What makes a unit? Beer: A premium pint of lager, bitter or cider (5%) contains 3 units

A ordinary strength pint of lager, bitter or cider (3-4%) contains 2 units

Wine: A large 250ml glass of wine (12%) contains 3 units, a standard 175ml glass of wine (12%) contains 2 units]

- 2 units or less per week
- 3-5 units per week
- 6-10 units per week
- 11-15 units per week
- 16 or more units each week

Do you have any physical health problems (not related to the pregnancy)? (Please describe)

Do you currently have any mental health problems? (Please describe)

Do you currently do any yoga or meditation?

- Yes
- No

What current yoga or meditation do you do (on average)? (select all that apply)

- Yoga once a month
- Yoga once every two weeks
- Yoga once a week
- Yoga more than once a week
- Yoga every day
- Meditation once a month
- Meditation once every two weeks
- Meditation every week
- Meditation a few times a week
- Meditation every day
- Other (please specify):

Are you currently going to any support groups?

- Yes
- No

What sorts of groups do you attend? (select all that apply)

- Baby group
- Childbirth Preparation, e.g. NCT
- Other (please specify):

Are you getting any support online?

- Yes
- No

What websites are you visiting for support or information at the moment? (select all that apply)

- National Childbirth Trust website
- National Childbirth Trust Forum
- Facebook Group/s
- NHS Choices
- Parenthood Forum
- Other (please specify):

If an online parenthood forum or Facebook group, which one/s? (Optional)
